# Supplementary material for: Lateral parabrachial FoxP2 neurons regulate respiratory responses to hypercapnia
Source: Nat Commun. 2024 May 25;15:4475. doi: 10.1038/s41467-024-48773-5 (PMC11128025; doi:10.1038/s41467-024-48773-5)
Supplement: Supplementary file 1 — Supplementary Information [file 41467_2024_48773_MOESM1_ESM.pdf]

Lateral parabrachial FoxP2 neurons regulate respiratory responses to hypercapnia

Satvinder Kaur[1], Nicole Lynch[1], Yaniv Sela[1], Janayna D Lima[1], Renner C Thomas[1], Sathyajit S Bandaru[1], Clifford B Saper[1]*

[1]Department of Neurology, Division of Sleep Medicine, and Program in Neuroscience, Beth Israel Deaconess Medical Center and Harvard Medical School, Boston, Massachusetts, 02215, USA

*- corresponding author

Supplementary Figures and Figure Legends

Inventory of Supplementary figures:

1. **Supplementary Figure 1** *Some KF^{CGRP} neurons also express FoxP2.*
2. **Supplementary Figure 2** *Validation of eutopic expression of Cre recombinase in FoxP2 neurons in the PB^{Foxp2} cells.*
3. **Supplementary Figure 3** *Fiber and endomicroscopic photometry of the PBcl^{FoxP2} neurons.*
4. **Supplementary Figure 4** *Cross-correlation analysis between ΔF/F of the PBcl^{FoxP2} neurons and EEG.*
5. **Supplementary Figure 5** *Cross-correlation analysis between the ΔF/F of the PBcl^{FoxP2} neurons vs respiration and movement (EMG).*
6. **Supplementary Figure 6** *Effects of photoactivation of PB^{FoxP2} neurons on respiration.*
7. **Supplementary Figure 7** *Effect of photoactivation of PB^{FoxP2} neurons on respiration during the wake state.*

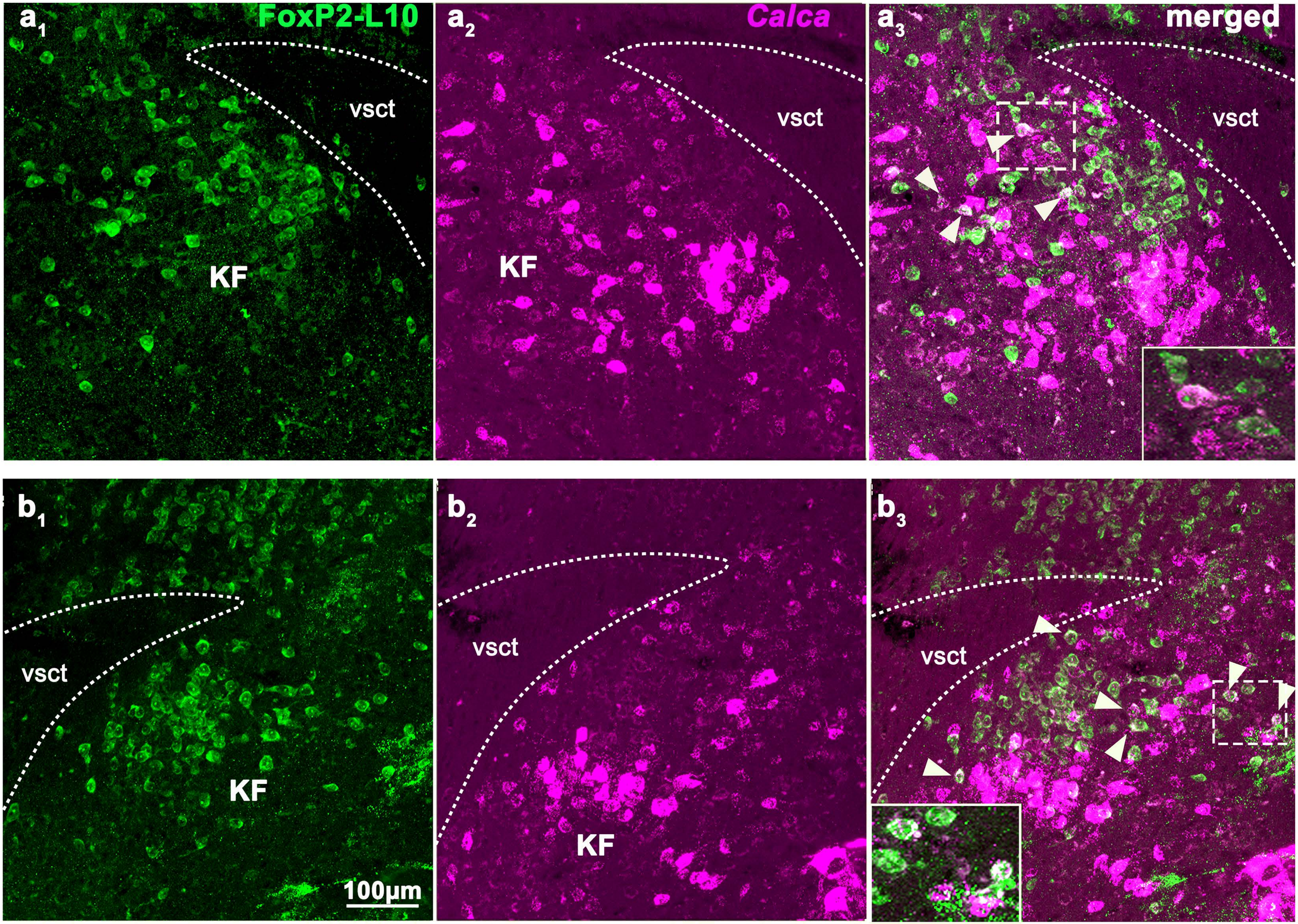
a1
FoxP2-L10
vsct
KF
a2
Calca
vsct
KF
a3
merged
vsct
b1
vsct
KF
100µm
b2
vsct
KF
b3
vsct

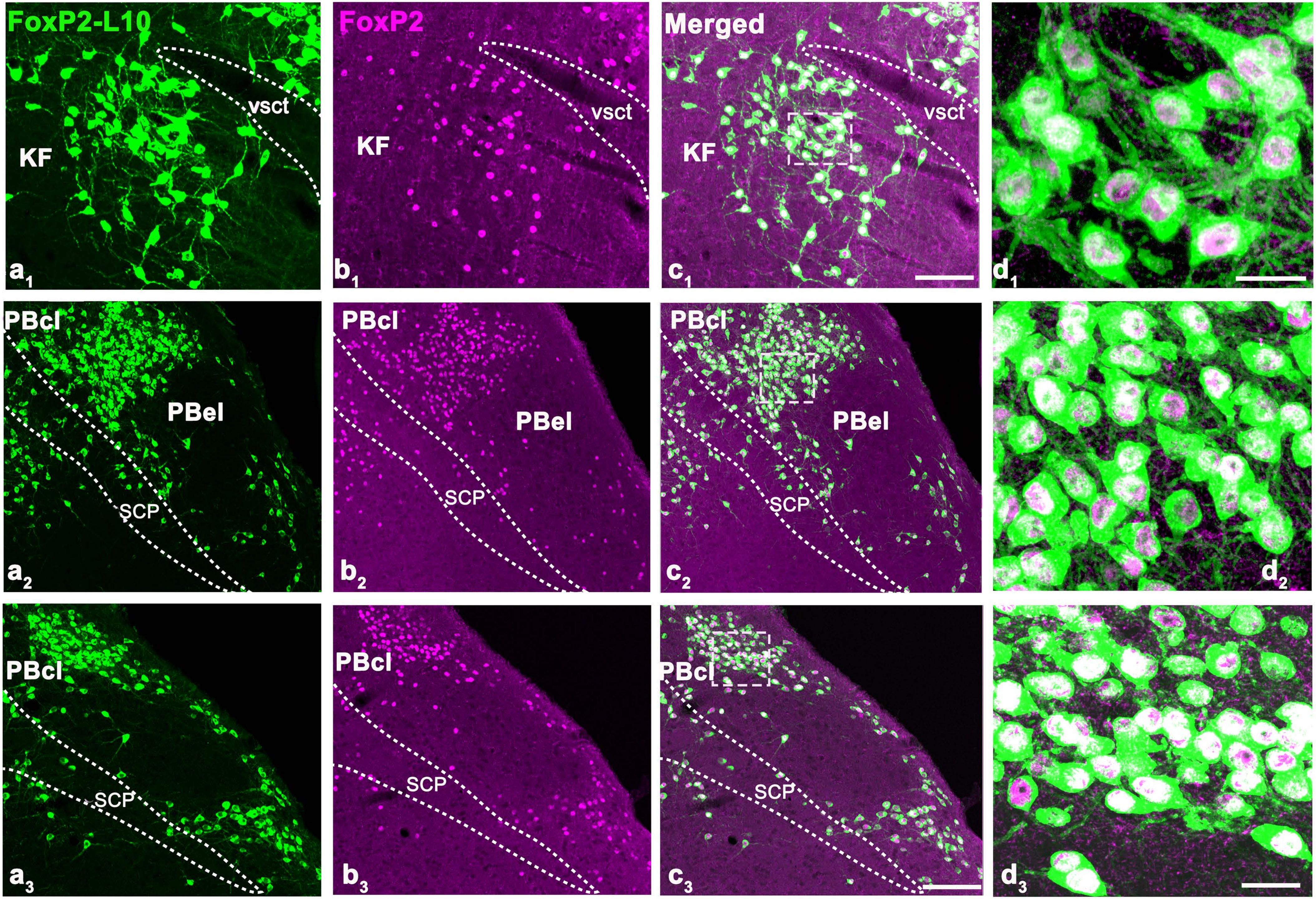

FoxP2-L10
FoxP2
Merged
KF
vsct
PBcl
PBel
SCP
a1
b1
c1
d1
a2
b2
c2
d2
a3
b3
c3
d3

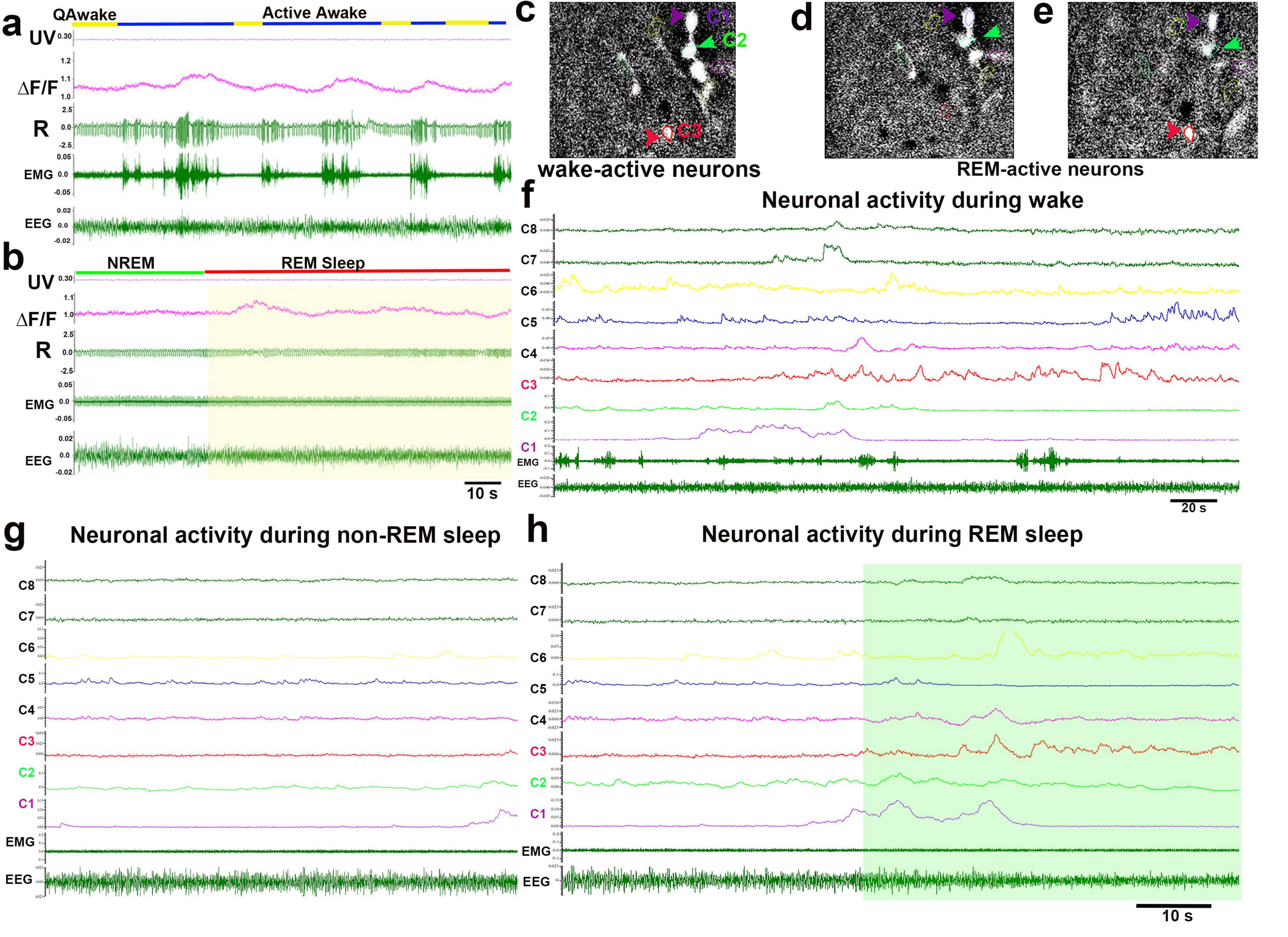

a
QAwake
Active Awake
UV
ΔF/F
R
EMG
EEG
b
NREM
REM Sleep
UV
ΔF/F
R
EMG
EEG
10 s
c
C1
C2
C3
wake-active neurons
d
e
REM-active neurons
f
Neuronal activity during wake
C8
C7
C6
C5
C4
C3
C2
C1
EMG
EEG
20 s
g
Neuronal activity during non-REM sleep
C8
C7
C6
C5
C4
C3
C2
C1
EMG
EEG
h
Neuronal activity during REM sleep
C8
C7
C6
C5
C4
C3
C2
C1
EMG
EEG
10 s

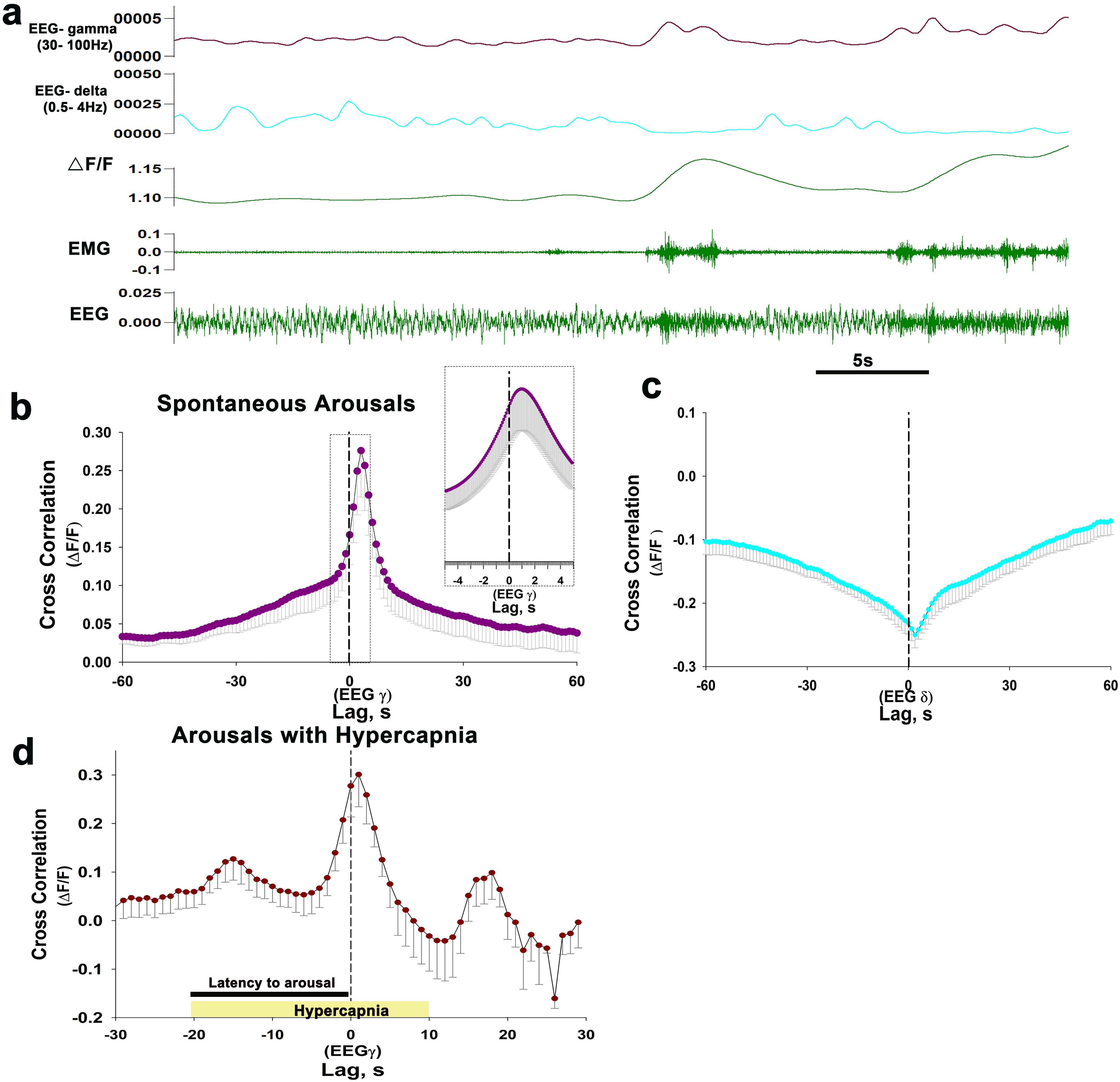

a
EEG- gamma (30- 100Hz)
00005
00000
EEG- delta (0.5- 4Hz)
00050
00025
00000
△F/F
1.15
1.10
EMG
0.1
0.0
-0.1
EEG
0.025
0.000
5s
b
Spontaneous Arousals
Cross Correlation (ΔF/F)
0.30
0.25
0.20
0.15
0.10
0.05
0.00
-60
-30
0
30
60
(EEG γ)
Lag, s
-4
-2
0
2
4
(EEG γ)
Lag, s
c
Cross Correlation (ΔF/F)
0.1
0.0
-0.1
-0.2
-0.3
-60
-30
0
30
60
(EEG δ)
Lag, s
d
Arousals with Hypercapnia
Cross Correlation (ΔF/F)
0.3
0.2
0.1
0.0
-0.1
-0.2
Latency to arousal
Hypercapnia
-30
-20
-10
0
10
20
30
(EEGγ)
Lag, s

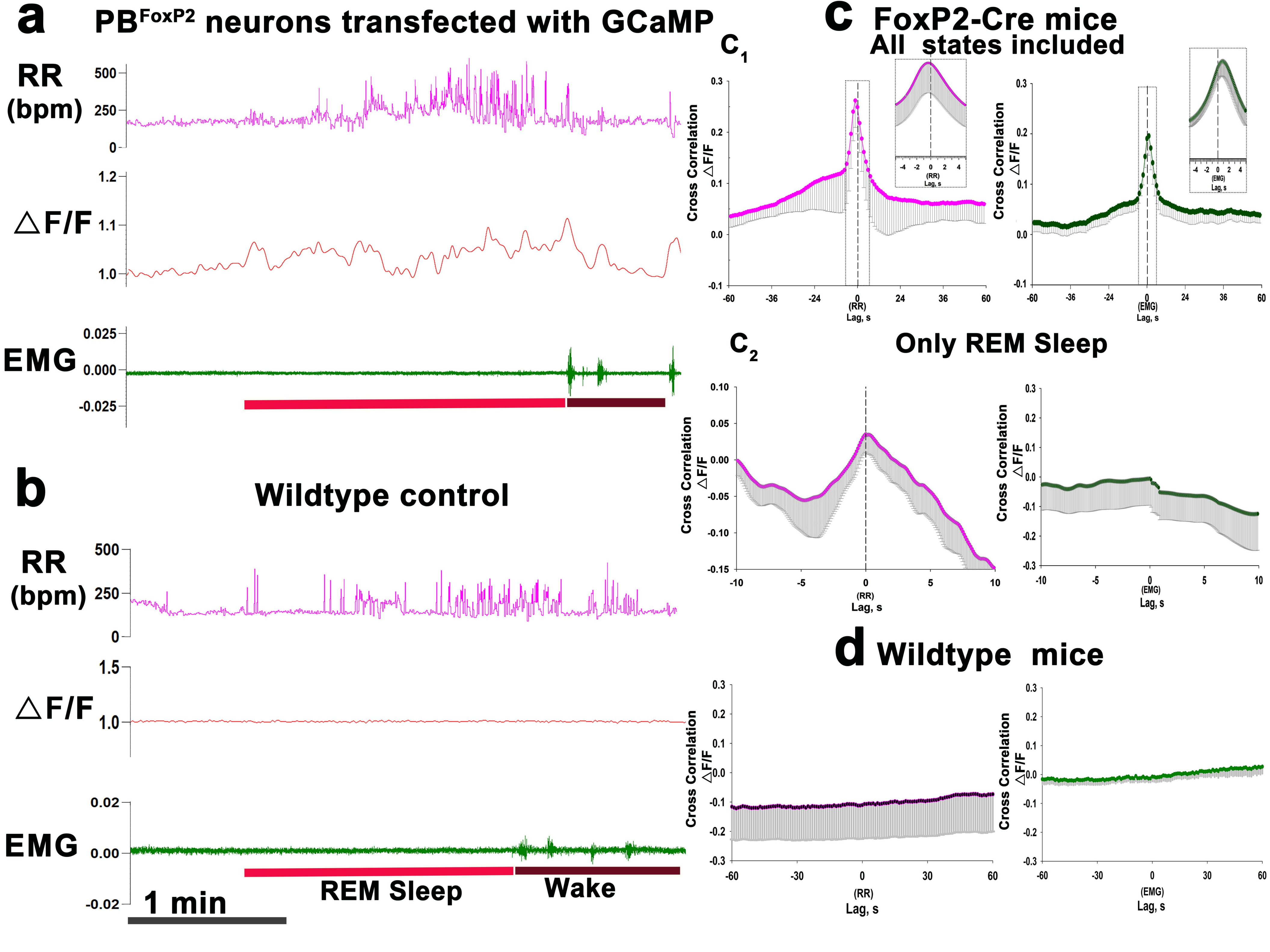
a
PB^FoxP2 neurons transfected with GCaMP
RR (bpm)
△F/F
EMG
b
Wildtype control
RR (bpm)
△F/F
EMG
1 min
REM Sleep
Wake
c
FoxP2-Cre mice
All states included
C1
Cross Correlation △F/F
(RR) Lag, s
(EMG) Lag, s
C2
Only REM Sleep
d
Wildtype mice

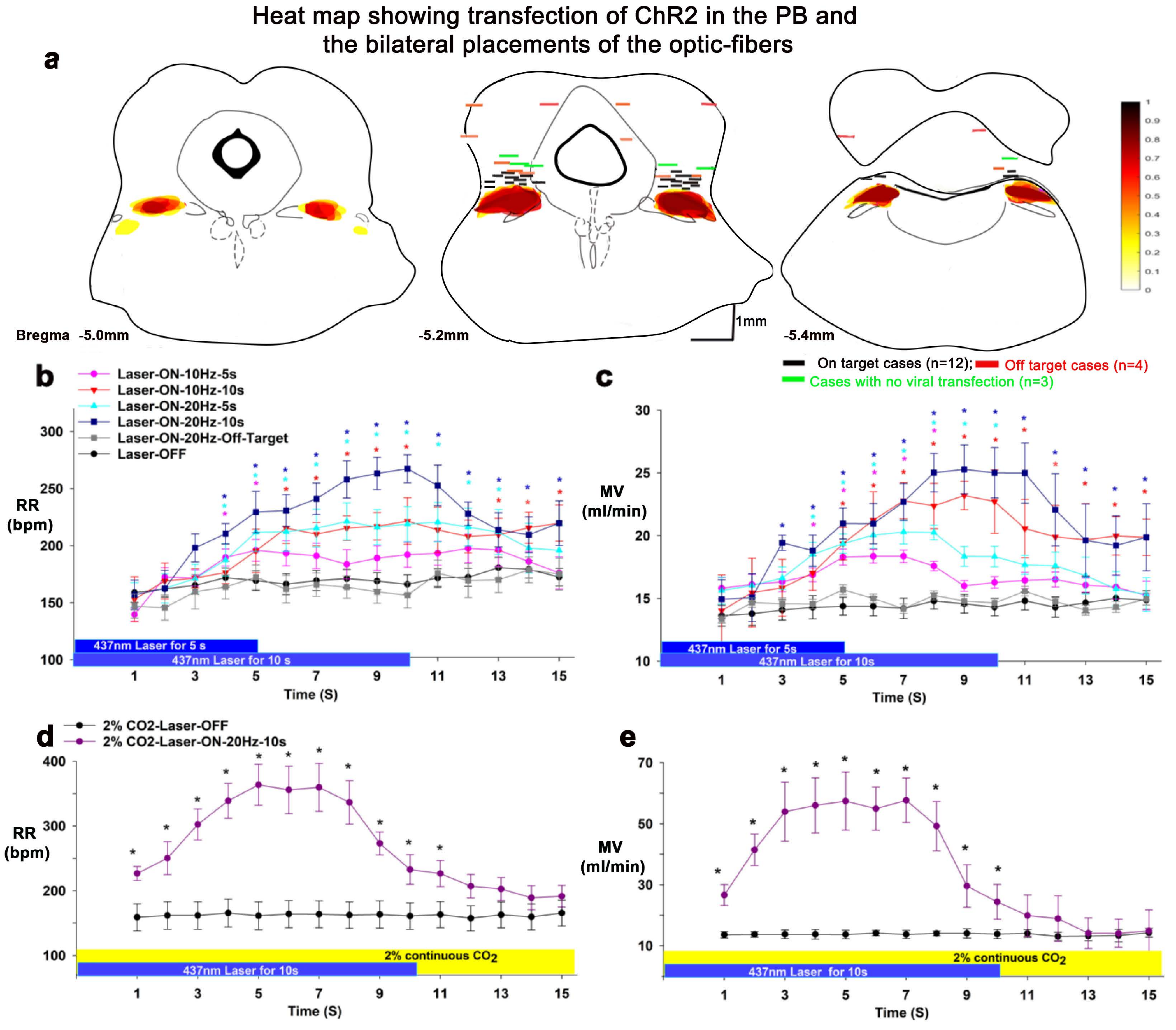
Heat map showing transfection of ChR2 in the PB and
the bilateral placements of the optic-fibers
a
Bregma -5.0mm
-5.2mm
-5.4mm
1mm
On target cases (n=12);
Off target cases (n=4)
Cases with no viral transfection (n=3)
b
Laser-ON-10Hz-5s
Laser-ON-10Hz-10s
Laser-ON-20Hz-5s
Laser-ON-20Hz-10s
Laser-ON-20Hz-Off-Target
Laser-OFF
RR
(bpm)
437nm Laser for 5 s
437nm Laser for 10 s
Time (S)
c
MV
(ml/min)
437nm Laser for 5s
437nm Laser for 10s
Time (S)
d
2% CO2-Laser-OFF
2% CO2-Laser-ON-20Hz-10s
RR
(bpm)
2% continuous CO2
437nm Laser for 10s
Time (S)
e
MV
(ml/min)
2% continuous CO2
437nm Laser for 10s
Time (S)

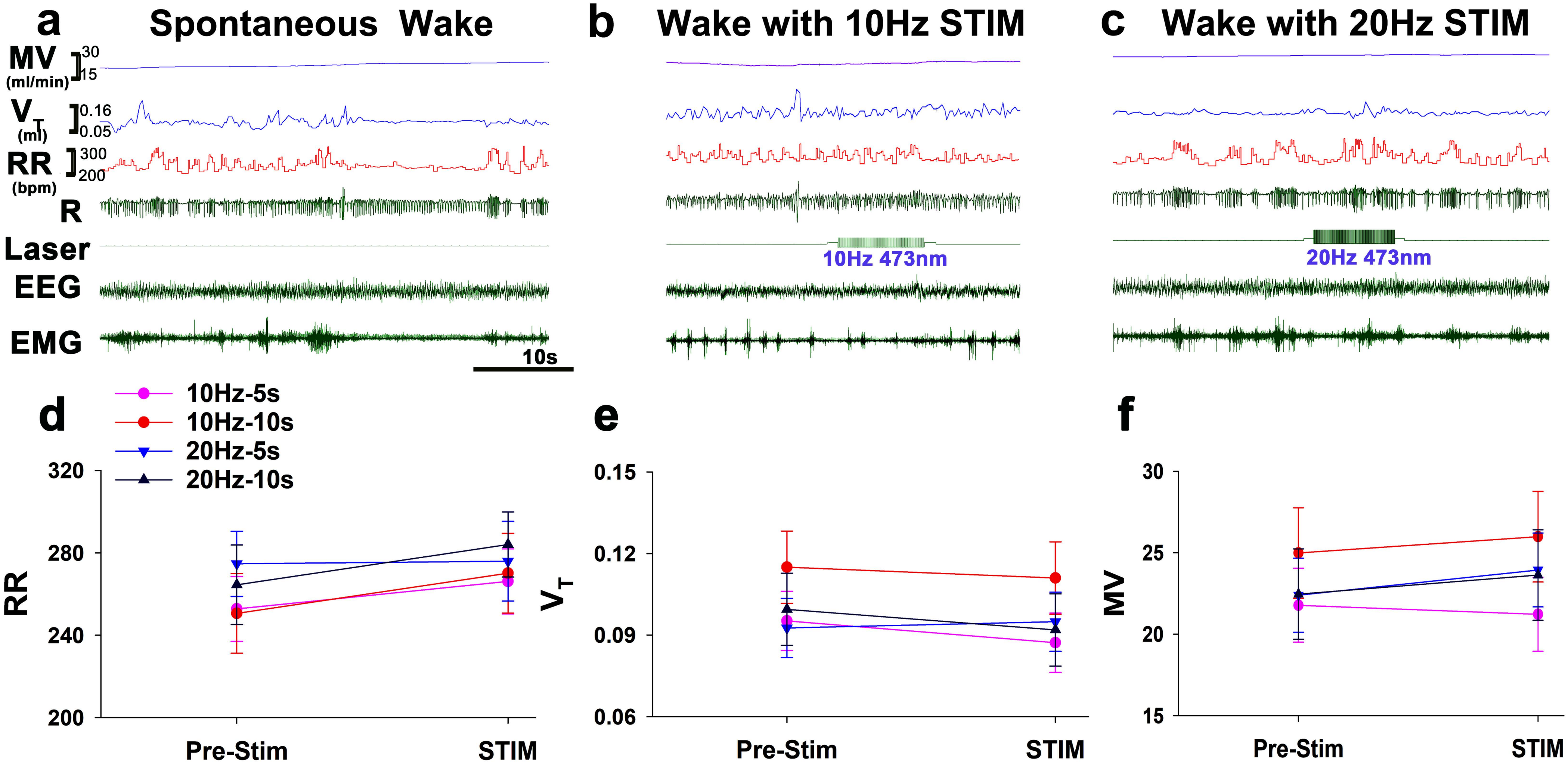
a
Spontaneous Wake
b
Wake with 10Hz STIM
c
Wake with 20Hz STIM
MV
(ml/min)
30
15
VT
(ml)
0.16
0.05
RR
(bpm)
300
200
R
Laser
EEG
EMG
10s
10Hz 473nm
20Hz 473nm
d
e
f
10Hz-5s
10Hz-10s
20Hz-5s
20Hz-10s
RR
320
280
240
200
VT
0.15
0.12
0.09
0.06
MV
30
25
20
15
Pre-Stim
STIM

Supplementary Figure legends:

Supplementary Figure 1 *Some KF^{CGRP} neurons also express FoxP2*: Photomicrographs of coronal brain sections showing FoxP2-L10 labeled neurons (green for GFP) at the level of the KF (**a1 and b1**) and double-labeled for *Calca* mRNA (the gene for CGRP), magenta, **a2** and **b2**) using RNA-scope, are from the right (**a1- a3**) and left (**b1-b3**) side of the brain. A small number of KF neurons were double-labeled for both FoxP2 and *Calca* (white) are marked by white arrowheads in **a3** and **b3**, and the insets show 2x magnified view of the areas marked by dash squares (n=3). These neurons were intermixed with other CGRP, non-FoxP2 neurons, which were slightly larger, and appeared to be a rostral continuation of the $PBel^{CGRP}$ population. Scale = 100µm.
Abbreviations; KF- Kölliker Fuse nucleus; vsct- ventral spino-cerebellar tract.

Supplementary Figure 2 *Validation of eutopic expression of Cre recombinase in FoxP2 neurons in the PB^{Foxp2} cells:* We validated the presence of the Cre-recombinase enzyme in adult male mice by crossing *$Foxp2^{tm1.1(cre)Rpa}$*/J transgenic mice with R26-lox-STOPlox-L10-GFP mice which labels the ribosomes of the cre expressing neurons with GFP. Brain sections (rostral to caudal levels 1, 2 and 3) were immunohistochemically stained for GFP (green, **a1-3**) and FoxP2 (magenta, **b1-3**) and overlap was compared at low (**c**) and high magnification (d). As FoxP2 is a transcription factor, immunostaining (magenta) was confined to the nucleus. The nuclei of all GFP labeled cell bodies in the KF and PB showed magenta FoxP2 immunostaining, and vice versa (n=3). Scale in c1= 80 µm; d1= 20 µm; c3= 100 µm; d3=18 µm.
Abbreviations: KF- Kölliker Fuse PB subnucleus; PBcl- central lateral PB subnucleus; PBel- external lateral PB subnucleus; scp- superior cerebellar peduncle; vsct- ventral spino-cerebellar tract.

Supplementary Figure 3 *Fiber and endomicroscopic photometry of the $PBcl^{FoxP2}$ neurons* showed increased GCaMP fluorescence ($\Delta F/F$) when mice (n=5) transitioned from NREM sleep either to the wake or the REM sleep state. With fiber photometry, peaks in increased activity during wake were associated with episodes of increased respiration (R) during active movement (**a**) compared to quiet wake (QAwake). Similar peaks were seen in REM sleep, without movement, but not in NREM sleep (**b**).
With endomicroscopy (**c-g**), 83% of the $PBcl^{FoxP2}$ neurons showed higher calcium activity during both REM and Wake, but the peaks in firing of individual neurons while still greater around the time of movements, did not align as precisely with the episodes of movement as did the population response (c.f., **a** vs. **f**). Representative examples are shown of the activity profiles of 8 neurons (including the 3 neurons shown in **c-e**, for which the traces in **f-h** are the same color as the arrows in (**c-e**) during wake with high motor activity (**f**), NREM sleep with little movement (g) and REM sleep (h) with no movement. The activity patterns of some individual PB^{FoxP2} neurons which were aligned with each other (e.g., **c1-4**) during wake were also aligned during REM sleep, suggesting that they may have been driven by fictive movements (generated by the forebrain, but blocked by the atonia circuitry) during REM dreams.

Supplementary Figure 4 *Cross-correlation analysis between $\Delta F/F$ of the $PBcl^{FoxP2}$ neurons and EEG*: A representative trace from a FoxP2-Cre mouse, showing EEG, EMG, $\Delta F/F$, and EEG power at gamma (γ, 30-100Hz) and delta (δ, 0.5- 4.0Hz) frequencies in **a**. Increased EEG δ power is indicative of NREM sleep, while increased EEGγ power indicates arousal/ wake.

Cross-correlation of ΔF/F with EEGγ power (mean ± SEM) during spontaneous arousals shows a positive correlation with the EEG arousal (t=0) which precedes the peak in ΔF/F by about 1s (sampling at 1Hz) (**b**) in n=5 mice. Inset in b is a magnified portion of marked area in the graph sampled at 10Hz resolution. Cross-correlation of ΔF/F with EEG δ (mean ± SEM), by contrast, indicates a negative correlation with ΔF/F following EEG δ indicating spontaneous arousal by about 1s (**c**). Cross-correlation during an arousal in response to hypercapnia demonstrates a smaller peak in ΔF/F about 5s after onset of the CO2 stimulus and about 15s before arousal which showed a much larger increase in ΔF/F that peaked about 1s after EEGγ (**d**). The yellow rectangle along the X-axis marks the period when the animal was exposed to hypercapnia in these trials (**d**).

Supplementary Figure 5 *Cross-correlation analysis between the ΔF/F of the $PBcl^{FoxP2}$ neurons vs respiration and movement (EMG)*: Representative traces from a FoxP2-Cre mouse (**a**) and a wildtype mouse (**b**, with no GCaMP expression), showing the respiratory rate (RR), GCaMP ΔF/F, and the EMG (representing activity/movement) during REM sleep (red bar) and wake states (dark brown bar). Cross correlation analysis (mean ± SEM) that included all behavioral states (including both sleep and wake) in the FoxP2-Cre mice (n=5) are shown in **c1**; while analysis that included only the REM sleep episodes, are shown in **c2**. The insets in **c1** are the magnified portion of the graphs sampled at 10Hz for better resolution. Similar analysis done in the wildtype mice with no GCaMP expression shows no correlation (**d**). Note that in general ΔF/F peaks before the onset of increased RR and at 1s after the onset of movement (EMG), but during REM sleep ΔF/F correlates with RR but not with EMG.

Supplementary Figure 6 *Effects of photoactivation of PB^{FoxP2} neurons on respiration:* A heat map (**a)** shows the extent of AAV-Flex-ChR2 transfection of the FoxP2 neurons in the PBcl region at three levels of bregma rostro-caudally (n=19). All animals had bilateral injections and placement of optical fibers. The location of on-target optical fiber placements are marked in black (n=12). Cases with off-target fiber placement (n=4) are marked in red and those with on-target fiber placement but lacking ChR2-transfected neurons in the area (n=3, off-target injection placement) are marked in green. No changes in respiration with laser stimulation (20Hz 10s) were seen in cases in which both optical fibers or both injections were off-target. The graphs in **b- e**, plot the instantaneous changes in RR and MV (mean ± SEM) in cases with bilateral accurate fiber placement, with either 10 or 20Hz laser (473nm) stimulation for either 5 or 10s in normocapnic air (**b, c**) or with continuous 2% CO2 in addition to the laser stimulus at 20Hz (**d, e**) during NREM sleep. Two-way ANOVA was used for statistical comparison, followed by Holms-Sidak test for multiple comparisons, where *= P<0.01 (vs. Laser OFF and Off-Target control); and color of * represents the comparison group (as shown in graph legend **in b**) to the controls. RR and MV rapidly increased throughout the laser stimulation and remained elevated with slow waning over about 10s after the laser was turned off. In 2% CO2, the RR and MV increased much more rapidly, peaking in less than 5s at a RR and MV about three times baseline, but began to wane after about 8s, even while the laser was still on.

Supplementary Figure 7 *Effects of photoactivation of PB^{FoxP2} neurons on respiration during the wake state:* Representative traces from a mouse during a spontaneous awake state without stimulation (**a**); and after 10Hz (**b**) and 20Hz (**c**) laser stimulation. The graphs comparing the respiratory rate (RR); tidal volume (V_T) and minute ventilation (MV) in the trials during which animals were awake are shown in **d-f**. Laser stimulation at either 10 or 20Hz (n=6) appeared to

cause a small increase in RR and MV, but this did not reach statistical significance. Thus, in the waking state, the contribution of the $PBcl^{FoxP2}$ neurons to ventilation appears not to be important, or they may already be firing at their maximal rate. Similarly, the photoinhibition also had no significant effects on respiration during either wake or REM sleep states (not shown).
