# Supplementary material for: Lateral parabrachial FoxP2 neurons regulate respiratory responses to hypercapnia
Source: Nat Commun. 2024 May 25;15:4475. doi: 10.1038/s41467-024-48773-5 (PMC11128025; doi:10.1038/s41467-024-48773-5)
Supplement: Supplementary file 3 — Description of Additional Supplementary Files [file 41467_2024_48773_MOESM3_ESM.pdf]

Description of Additional Supplementary Files:

Supplementary Movie 1: Wake-active neurons.mp4: Representative video showing the activity profiles of 4 parabrachial FoxP2 wake-active neurons (top left), which show peak fluorescence (higher Ca_i) during the active awake state. The colored arrows (matching the color of their activity profile curves) in the top right panel point to the active neurons (DF/F) during movement (higher EMG) shown in the lower panel marked locomotor activity.

Supplementary Movie 2: REM-active neurons.mp4: Representative video showing the activity profiles of the same 4 neurons that were shown in SVideo1 (Wake-active neurons), are also seen to be active during REM sleep, when there is no EMG activity (as shown in the lower panel- locomotor activity).

Supplementary Movie 3: CO2 responsive neurons.mp4: Representative video showing neuronal activity profiles from 4 parabrachial FoxP2 neurons that showed peaks in calcium fluorescence during exposure to CO2. The upper left panel shows the CO2 levels in the chamber and the upper right shows raw GCaMP fluorescence imaging. The panel on the lower right shows the same field, thresholded to show change in fluorescent signal (DF/F). The calcium signal from four individual neurons (color coded for the neurons encircled in the lower right panel) is plotted across time in synchrony with the CO2 curve, in the lower left panel. The colored arrows (color matched their activity profile curves) in the bottom right panel show the activated neurons (DF/F) and are seen for the duration of their activation.
