# Supplementary material for: Lateral parabrachial FoxP2 neurons regulate respiratory responses to hypercapnia
Source: Nat Commun. 2024 May 25;15:4475. doi: 10.1038/s41467-024-48773-5 (PMC11128025; doi:10.1038/s41467-024-48773-5)
Supplement: Supplementary file 7 — Reporting Summary [file 41467_2024_48773_MOESM7_ESM.pdf]

Corresponding author(s): Clfford B. Saper
Last updated by author(s): 04/12/2024

Reporting Summary

Nature Portfolio wishes to improve the reproducibility of the work that we publish. This form provides structure for consistency and transparency in reporting. For further information on Nature Portfolio policies, see our Editorial Policies and the Editorial Policy Checklist.

Statistics

For all statistical analyses, confirm that the following items are present in the figure legend, table legend, main text, or Methods section.

n/a	Confirmed	
☐	☒	The exact sample size (*n*) for each experimental group/condition, given as a discrete number and unit of measurement
☐	☒	A statement on whether measurements were taken from distinct samples or whether the same sample was measured repeatedly
☐	☒	The statistical test(s) used AND whether they are one- or two-sided<br>*Only common tests should be described solely by name; describe more complex techniques in the Methods section.*
☐	☒	A description of all covariates tested
☐	☒	A description of any assumptions or corrections, such as tests of normality and adjustment for multiple comparisons
☐	☒	A full description of the statistical parameters including central tendency (e.g. means) or other basic estimates (e.g. regression coefficient) AND variation (e.g. standard deviation) or associated estimates of uncertainty (e.g. confidence intervals)
☐	☒	For null hypothesis testing, the test statistic (e.g. *F*, *t*, *r*) with confidence intervals, effect sizes, degrees of freedom and *P* value noted<br>*Give P values as exact values whenever suitable.*
☒	☐	For Bayesian analysis, information on the choice of priors and Markov chain Monte Carlo settings
☒	☐	For hierarchical and complex designs, identification of the appropriate level for tests and full reporting of outcomes
☒	☐	Estimates of effect sizes (e.g. Cohen's *d*, Pearson's *r*), indicating how they were calculated

Our web collection on statistics for biologists contains articles on many of the points above.

Software and code

Policy information about availability of computer code

Data collection	software:<br>1. Axoscope software- v10 (Molecular Devices, Foster City, CA, USA)<br>2. Spike2 ver.7 (CED, Cambridge, UK).<br>3. Calcium imaging using Inscopix Data acquisition software
Data analysis	1. Analysis of calcium imaging using the Inscopix data processing (IDPS ver1.6).<br>2. Respiratory and EEG data analysis- Spike2 ver.7 (CED, Cambridge, UK).<br>3. Spike respiratory scripts (Resp80t, Spike2, CED, UK).<br>4. Statistical analyses and graph plotting using SigmaPlot 14.5 (Systat Software, Inc.).<br>5. Matlab (R2021a) for generation of heat maps.

For manuscripts utilizing custom algorithms or software that are central to the research but not yet described in published literature, software must be made available to editors and reviewers. We strongly encourage code deposition in a community repository (e.g. GitHub). See the Nature Portfolio guidelines for submitting code & software for further information.

Data

Policy information about availability of data

All manuscripts must include a data availability statement. This statement should provide the following information, where applicable:

- Accession codes, unique identifiers, or web links for publicly available datasets
- A description of any restrictions on data availability
- For clinical datasets or third party data, please ensure that the statement adheres to our policy

Source data are provided with this paper as "Source data file". All data generated to support the findings of this study are also available from the corresponding author upon reasonable request.

Research involving human participants, their data, or biological material

Policy information about studies with human participants or human data. See also policy information about sex, gender (identity/presentation), and sexual orientation and race, ethnicity and racism.

Reporting on sex and gender	N/A
Reporting on race, ethnicity, or other socially relevant groupings	N/A
Population characteristics	N/A
Recruitment	N/A
Ethics oversight	N/A

Note that full information on the approval of the study protocol must also be provided in the manuscript.

Field-specific reporting

Please select the one below that is the best fit for your research. If you are not sure, read the appropriate sections before making your selection.

☒ Life sciences ☐ Behavioural & social sciences ☐ Ecological, evolutionary & environmental sciences

For a reference copy of the document with all sections, see nature.com/documents/nr-reporting-summary-flat.pdf

Life sciences study design

All studies must disclose on these points even when the disclosure is negative.

Sample size	Using SigmaPlot 14.5, we tested the sample size and power of the tests post hoc and found that the power of each statistical test was at least 80% at alpha= 0.05, suggesting adequate sample sizes for all the experiments.
Data exclusions	No data was excluded.
Replication	All physiology experiments were replicated many times (trials) in same animal, consistency and variability of data as per the site manipulated has been discussed in the results section. Similarly, immuno-histochemistry data represented as photomicrograph are consistency reproduced in "n" number of animals mentioned in legends of each of these figures.
Randomization	Randomization was adopted for the treatments (laser-On vs. laser-off and for different parameters of Laser stimulation)
Blinding	To prevent bias, individuals blinded to the treatment groups analyzed histology, cell counts and respiratory data analysis, and this is mentioned throughout the manuscript at appropriate places. Blinding to the experimental groups was done after data collection, as correct experimental parameters cannot be applied blindly by individuals involved in data collection. However, the individuals involved in data collection were not involved in data analysis.

Reporting for specific materials, systems and methods

We require information from authors about some types of materials, experimental systems and methods used in many studies. Here, indicate whether each material, system or method listed is relevant to your study. If you are not sure if a list item applies to your research, read the appropriate section before selecting a response.

Materials & experimental systems

n/a	Involved in the study
☐	☒ Antibodies
☒	☐ Eukaryotic cell lines
☒	☐ Palaeontology and archaeology
☐	☒ Animals and other organisms
☒	☐ Clinical data
☒	☐ Dual use research of concern
☒	☐ Plants

Methods

n/a	Involved in the study
☒	☐ ChIP-seq
☒	☐ Flow cytometry
☒	☐ MRI-based neuroimaging

Antibodies

Antibodies used

1. c-Fos antibody (Oncogene Sciences, cat # Ab5 was a rabbit polyclonal, antiserum raised against amino acids 4–17 of human c-Fos) dilution (1:10K).
2. Sheep anti-FoxP2 (R and D Systems Cat# AF5647, RRID:AB_2107133), dilution- 1:5K.
3. Biotinylated secondary antibody (Donkey anti-sheep- biotinylated, dilution-1:200, Cat#-713-065-147; RRID: AB_2340716; Jackson-immuno Research)
4. Streptavidin-conjugated Cy3 (red fluorochrome) (dilution-1:200, ThermoFischer, Cat#- 434315).
5. GFP (Rabbit anti-GFP, dilution- 1:10K, Molecular Probes Cat# A-11122, RRID:AB_221569).
6. mCherry (Rabbit anti DsRed. dilution-1:2K, Clontech, Cat-632496)
7. Goat anti-CTb (dilution-1:30K, Cat# 703, RRID: AB_10013220, List Biological Laboratories Inc., CA).
8. Rabbit anti-Foxp2, (dilution-1:10K, Abcam Cat# ab16046, RRID:AB_2107107)
9. Fluorescent-labeled secondary antibodies donkey anti goat- Alexa- 555 (red) at dilution-1:200 (Cat # A-21432, RRID- AB_2535853) or donkey anti rabbit- Alexa-488 (green) at duilution-1:200 (Cat # A-21206, and RRID-AB_2535792) (Molecular probes, Thermo-Fischer Scientific).

Validation

1. cFos antibody- This antiserum stained a single band of 55 kDa on Western blots from rat brain (manufacturer's technical information).
2. Sheep anti-FoxP2 (R and D Systems Cat# AF5647, RRID:AB_2107133) is a polyclonal antiserum is raised against recombinant human FoxP2 isoform 1, Ala640-Glu715 (accession # O15409), shown by the manufacturer to be specific for human and mouse, shows a single band for FoxP2 at approximately 80 kDa, and is also previously used by others 59–61.
3. Rabbit polyclonal antibody to FoxP2 was raised against a synthetic peptide made from residues 700 to the C-terminus of human FoxP2 which was conjugated to keyhole limpet hemocyanin.This antibody also showed a single band in Western blots performed by the manufacturers and were specific to human and mouse FoxP2.

Animals and other research organisms

Policy information about studies involving animals; ARRIVE guidelines recommended for reporting animal research, and Sex and Gender in Research

Laboratory animals

Foxp2tm1.1(cre)Rpa/J transgenic mice originally prepared by Dr. Richard Palmiter, University of Washington and obtained from Jackson Laboratories, in which IRES-Myc tag-nuclear localization signal (NLS)-cre-GFP-frt-neomycin-frt were introduced just after the termination codon of the mouse Foxp2 gene via homologous recombination. The mutation was created via homologous recombination in (129S6/SvEvTac x C57BL/6) F1-derived G4 embryonic stem (ES) cells. The frt-flanked neomycin cassette was excised through crosses with animals that broadly expressing Flp recombinase. The GFP is believed to be nonfunctional. Resultant mice were backcrossed to C57BL/6J for 9 generations by the donating laboratory to the Jackson laboratory (Strain #:030541; RRID:IMSR_JAX:030541). All mice used in the study were at least 8-10weeks old at the time of surgery.

Wild animals

No wild animals were used in the study due to lack of appropriate controls and larger variability in them.

Reporting on sex

All mice used in these experiments were male because female mice of the same age are smaller, and including animals of various sizes would introduce noise into analysis of respiratory volumes (which scale with body size) across groups.

Field-collected samples

No field collected samples were used in the study.

Ethics oversight

All animal procedures met National Institutes of Health standards, as described in the Guide for the Care and Use of Laboratory Animals, and all protocols were approved by the Beth Israel Deaconess Medical Center Institutional Animal Care and Use Committee.

Note that full information on the approval of the study protocol must also be provided in the manuscript.

Plants

Seed stocks	N/A
Novel plant genotypes	N/A
Authentication	N/A
